# Knowledge, attitude, and practice regarding brucellosis among confirmed cases

**DOI:** 10.1038/s41598-025-30473-9

**Published:** 2025-11-28

**Authors:** Lingling Wang, Peipei Lu, Xuhong Wang, Hui Fan, Xiaoyi Zhang, Hui Rong, Xia Han, Meina Hu, Pinjian Ji, Feifei Chang, Zhiwei Li

**Affiliations:** 1 Clinical Laboratory Center, People’s Hospital of Xinjiang Uygur Autonomous Region, Urumqi, 830001 Xinjiang China; 2 Clinical Laboratory Department, Shawan People’s Hospital, Shawan, 832100 Xinjiang China; 3 Department of Laboratory Medicine, Wusu People’s Hospital, WuSu, 833000 Xinjiang China; 4 Clinical Laboratory, The First People’s Hospital of Korla, Korla, 841000 Xinjiang China; 5 Department of Medical Laboratory Science, People’s Hospital of Bayingolin Mongol Autonomous Prefecture, Korla, 841000 Xinjiang China; 6 Department of Clinical Laboratory, People’s Hospital of Altay Prefecture, Altay, 836400 Xinjiang China; 7 Clinical Laboratory Center, People’s Hospital of Kizilsu Kirghiz Autonomous Prefecture, Artux, 845350 Xinjiang China; 8 Department of Clinical Laboratory, Xinhua Hospital, Ili Kazak Autonomous Prefecture, Yining, 835000 Xinjiang China

**Keywords:** Brucellosis, Patients, Knowledge, attitudes, and practices, Cross-Sectional study, Health education, Disease prevention, Diseases, Medical research

## Abstract

Brucellosis remains a major public health problem in many endemic regions of China, particularly in areas with extensive livestock farming. This study aims to explore the knowledge, attitudes, and practices regarding brucellosis among patients with the disease. The cross-sectional study was conducted in Xinjiang from June 1, 2024, to January 30, 2025. Data were collected through a self-designed questionnaire encompassing sociodemographic characteristics and three dimensions of knowledge, attitudes, and practices. Of the 507 questionnaires distributed, 398 were valid and included in the analysis. Most respondents were male (62.81%) and worked in occupations involving contact with animals (65.08%). The mean scores for knowledge, attitude, and practice were 8.45 ± 3.06, 37.81 ± 3.89, and 38.62 ± 5.47, respectively. Structural equation modeling indicated that knowledge was positively associated with both attitudes (β = 0.579, *p* < 0.001) and practices (β = 0.351, *p* < 0.001), while attitudes also influenced practices (β = 0.270, *p* = 0.002). Knowledge further affected practices indirectly through attitudes (β = 0.156, *p* = 0.010). Brucellosis patients demonstrated relatively low knowledge scores but exhibited positive attitudes and proactive practices regarding the disease. The Structural equation modeling indicated that knowledge was associated with both attitudes and practices. Providing targeted educational interventions could help strengthen positive attitudes and improve preventive practices among brucellosis patients, ultimately supporting better disease control and management. In conclusion, strengthening patient-centered education and integrating it into routine public health programs are essential steps toward reducing the burden of brucellosis in endemic regions.

**Supplementary Information:**

The online version contains supplementary material available at 10.1038/s41598-025-30473-9.

## Introduction

Brucellosis, a widespread zoonotic disease caused by bacteria of the genus *Brucella*, continues to pose significant public health challenges globally, particularly in developing countries with extensive animal husbandry^1^. In China, the incidence of human brucellosis has risen markedly, from 0.92 cases per 100,000 people in 2004 to nearly 5 cases per 100,000 in 2023, reflecting a persistent upward trend^2–5^. Globally, the annual incidence of human brucellosis is estimated to be between 1.6 and 2.1 million new cases, with the highest burden in Asia and Africa^6^.In rural communities where animal husbandry is prevalent, insufficient health education combined with occupational exposure creates significant barriers to effective prevention and control^7^.

The disease is primarily transmitted from livestock to humans through direct contact or consumption of unpasteurized animal products. Xinjiang remains one of the key endemic areas in China^1,8–10^. However, while many studies have focused on farmers, herdsmen, or veterinarians, little is known about how patients themselves perceive and manage the disease after diagnosis, which represents an important knowledge gap this study aims to address. Understanding patients’ perspectives through the KAP framework is particularly valuable, as it provides structured insight into how knowledge influences attitudes and how these, in turn, shape preventive practices. The Knowledge, Attitudes, and Practices (KAP) survey functions as a diagnostic research tool, illuminating a group’s comprehension, beliefs, and actions on a specific subject, particularly within the realm of health literacy, where it is based on the premise that knowledge positively influences attitudes, which in turn mold behaviors^11^. Previous KAP studies on brucellosis have primarily focused on high-risk populations such as livestock farmers and veterinarians^12,13^. This research gap is significant, as patient understanding after diagnosis can affect treatment adherence and community transmission, highlighting the need to investigate patients’ KAP to inform targeted interventions, particularly given the risk of foodborne transmission through unpasteurized milk consumption^7,14^.

Therefore, this study investigates the KAP of brucellosis patients in China to provide evidence for improving health education strategies and public health policies. Therefore, this study investigates the KAP of brucellosis patients in China to provide evidence for improving health education strategies and public health policies.

## Methods

### Study design and participants

**Fig. 1:**
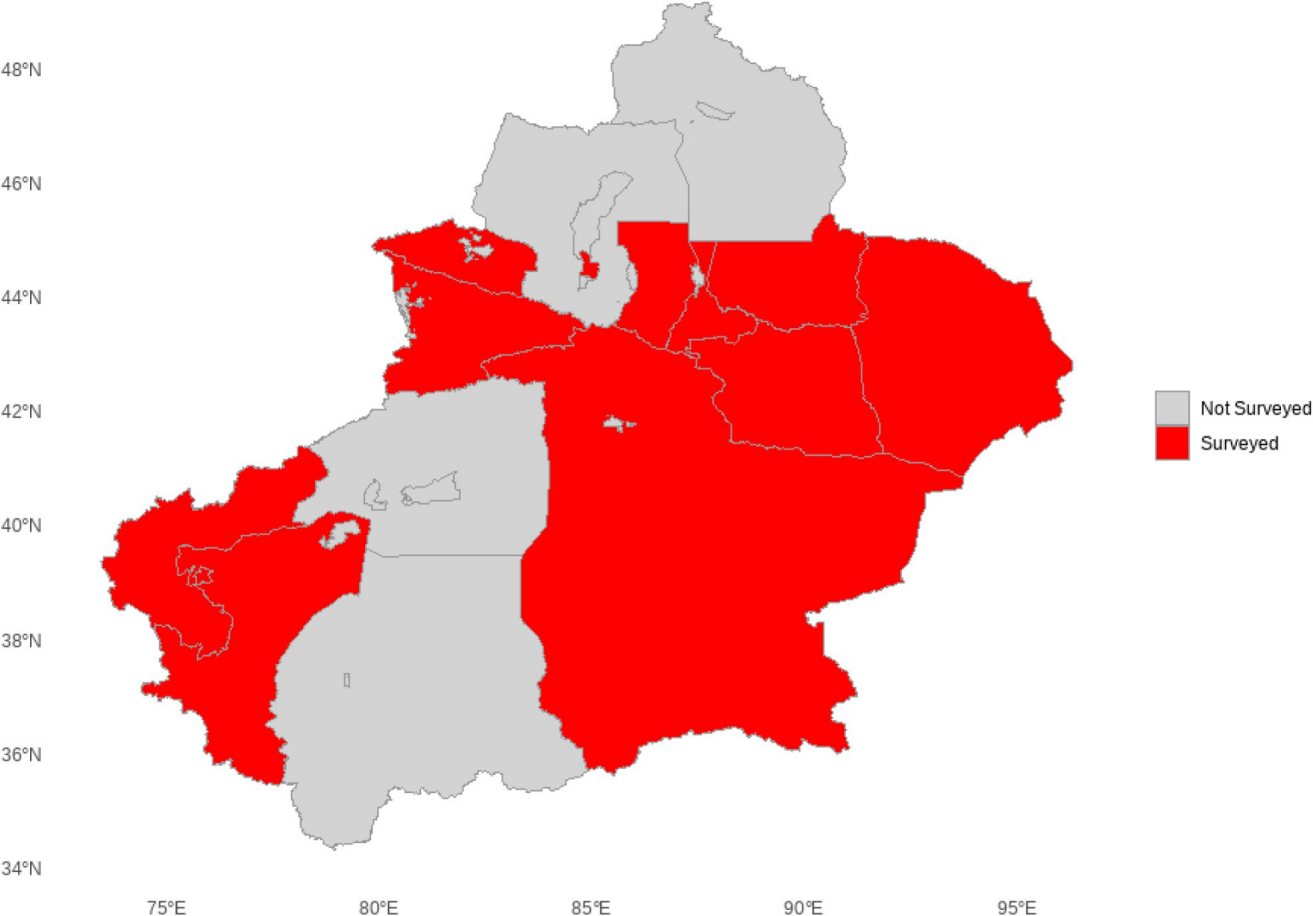
Study regions in Xinjiang covered by the brucellosis questionnaire survey.

The study population comprised laboratory-confirmed brucellosis patients from six prefectures in Xinjiang. This cross-sectional study was conducted in Xinjiang from June 1, 2024, to January 30, 2025, targeting patients with a confirmed history of brucellosis from Urumqi, Ili Kazakh Autonomous Prefecture, Changji Hui Autonomous Prefecture, Tacheng Prefecture, Bayingolin Mongol Autonomous Prefecture, and Altay Prefecture. Xinjiang was selected as the study area because it is one of the main endemic regions of brucellosis in China, where a large proportion of the population is engaged in pastoral and livestock-related occupations, resulting in a higher risk of infection. This study was approved by the Ethic Committee of People’s Hospital of Xinjiang Uygur Autonomous Region (KY2024053102), and all participants provided informed consent before enrollment. Patients were excluded if they were under 18 years of age, declined to participate, or completed the questionnaire in less than 30 s, as these responses were considered unreliable. Individuals younger than 18 years were excluded because their occupational exposure and health education levels differ substantially from adults, which could bias the assessment of knowledge, attitudes, and practices. A convenience sampling method was applied to recruit eligible participants, and a map of the study area is provided **(**Fig. 1**)**.

## Questionnaire

The questionnaire was designed based on references^15,16^. To evaluate its reliability, a pilot study with 56 participants was conducted, resulting in a Cronbach’s α coefficient of 0.864, which indicates good internal consistency.

The final version of the questionnaire, created in Chinese, included four sections: demographic information, knowledge, attitudes, and practices (**Appendix**). The demographic section gathered data on participants’ age, gender, marital status, place of residence, education level, and other relevant characteristics. The knowledge section contained 10 questions designed to assess participants’ awareness of brucellosis. Responses were categorized into three levels: “Very familiar,” “Heard of it,” and “Unclear,” corresponding to scores of 2, 1, and 0. Correct answers received a score of 2, while incorrect responses were scored as 0, resulting in a total possible score between 0 and 20. The attitude section included 9 questions rated on a five-point Likert scale, from “Strongly agree” (5 points) to “Strongly disagree” (1 point), with a total score range of 9 to 45. The practices section also contained 9 questions, with response options ranging from “Always” (5 points) to “Never” (1 point), leading to a total possible score between 9 and 45. To evaluate respondents’ levels of knowledge, attitudes, and practices, continuous scores were analyzed instead of applying a fixed threshold. This approach avoids arbitrary dichotomization and allows a more precise interpretation of variations across participants. The mean and distribution of scores in each KAP dimension were used to describe knowledge, attitudes, and practices comprehensively.

## Questionnaire distribution

The electronic questionnaire was created using the Wenjuanxing platform, which provided a QR code for accessing the survey. Search for the contact information of patients diagnosed with brucellosis in each hospital’s case system. Then, contact them by phone to ask them to scan the QR code and answer questions in the hospital consultation room. To improve data quality and ensure all responses were complete, each IP address was limited to a single submission, and all questions were marked as mandatory. Furthermore, the research team systematically reviewed all responses to verify their completeness, assess internal consistency, and confirm logical validity.

## Sample size

Sample size was determined using the standard formula for cross-sectional surveys. According to the formula for calculating the sample size in cross-sectional surveys . In the formula, “n” represents the sample size for each group, “α” represents the type I error, which is typically set at 0.05, = 1.96, δ represents the allowable error, typically set at 0.05, and “p” is set at 0.5 (as setting it at 0.5 maximizes the value and ensures a sufficiently large sample size). The calculated sample size “n” is 384, which is consistent with previous cross-sectional KAP studies that adopted a similar estimation approach^17^.

### Statistical analysis

Statistical analyses were conducted using the Statistical Package for the Social Sciences (SPSS) version 27.0 (IBM Corp., Armonk, NY, USA) and Analysis of Moment Structures (AMOS) version 26.0 (IBM Corp., Armonk, NY, USA). Continuous variables were assessed for normality using the Kolmogorov-Smirnov test and were expressed as means with standard deviations (SD) for normally distributed data or medians with interquartile ranges (IQR) for non-normally distributed data. Categorical variables were reported as frequencies and percentages (n, %). Comparisons between two groups were conducted using independent sample t-tests for normally distributed continuous variables and the Wilcoxon-Mann-Whitney test for non-normally distributed variables. For comparisons involving three or more groups, analysis of variance (ANOVA) was applied to normally distributed variables with homogeneity of variance, while the Kruskal-Wallis test was used for non-normally distributed data. Correlation analyses were performed using Pearson’s correlation coefficient for normally distributed variables and Spearman’s rank correlation coefficient for non-normally distributed variables. Univariate and multivariate liner regression analyses were conducted to identify factors associated with practices. Knowledge and attitude were included in the model as continuous variables. Multivariate liner regression was applied after correlation analysis to further determine independent predictors of practice scores while controlling for potential confounding variables. The β and 95% confidence intervals (CI) were reported to quantify the strength of associations. To examine the interrelationships among KAP, structural equation modeling (SEM) was applied. While correlation analysis was used to assess the strength and direction of pairwise associations between KAP variables, SEM employed to test the hypothesized causal pathways among knowledge, attitudes, and practices within a unified model. The hypothesized pathways included (1) a direct effect of knowledge on attitudes, (2) a direct effect of attitudes on practices, and (3) both direct and indirect effects of knowledge on practices through attitudes. In addition, a confirmatory factor analysis (CFA) was performed to validate the construct of the questionnaire. Model fit was assessed using the root mean square error of approximation (RMSEA), incremental fit index (IFI), Tucker-Lewis index (TLI), and comparative fit index (CFI), with acceptable thresholds based on conventional SEM criteria. All statistical tests were two-sided, with a significance level set at *P* < 0.05. Model fit was considered acceptable with RMSEA < 0.08, IFI > 0.80, TLI > 0.80, and CFI > 0.80.

## Results

### Baseline characteristics and KAP score

Initially, a total of 507 questionnaires were collected for this study. However, the following data were excluded: (1) 2 cases that did not consent to participate; (2) 15 cases from individuals under 18 years old; and (3) 92 cases with inconsistent responses. This left 398 valid cases for analysis. Among these participants, 250 (62.81%) were male, with a mean age of 47.56 ± 13.07 years. Additionally, 271 (68.09%) lived in rural areas, 183 (45.98%) resided in pastoral areas, 259 (65.08%) had occupations involving contact with animals, 284 (71.36%) reported a monthly per capita income of less than 5000 Yuan, and 112 (28.14%) included raw beef and mutton in their daily diets (Table 1). The Kaiser-Meyer-Olkin (KMO) value was 0.867, while RMSEA was 0.060, TLI was 0.829, IFI was 0.846, and CFI was 0.845, all indicating an acceptable model fit.

**Table 1:** Demographic characteristics.

	*n* (398)
**Age**,** years**	47.56 ± 13.07
**Gender**	
Male	250(62.81%)
Female	148(37.19%)
**Residence**	
Urban	127 (31.9%)
Rural	271(68.09%)
**Pastoral area residence**	
Yes	183(45.98%)
No	215(54.02%)
**Occupations involving contact with animals**	
Yes	259(65.08%)
No	139(34.92%)
**Education**	
Primary school or below	118(25.65%)
Junior high school	109 (27.39%)
Senior high school/technical secondary school	59 (14.82%)
Associate degree	53 (13.32%)
Bachelor’s degree or above	59(14.71%)
**Monthly income per capita**	
< 5000	284 (71.36%)
5000–10,000	95 (23.87%)
> 10,001	19 (4.74%)
**Raw beef or mutton in daily diet**	
Yes	112 (28.14%)
No	286 (71.86%)

The mean ± SD scores for knowledge, attitudes, and practices were 8.45 ± 3.06, 37.81 ± 3.89, and 38.62 ± 5.47, respectively. Knowledge scores were significantly higher among participants living in pastoral areas (*p* = 0.033), with higher education (*p* < 0.001), and higher income levels (*p* < 0.001). Attitudes scores also varied by pastoral area residence (*p* = 0.005) and dietary habits (*p* = 0.005). Furthermore, practices scores showed significant differences across residence (*p* < 0.001), pastoral area residence (*p* = 0.003), occupation involving animal contact (*p* = 0.009), education (*p* = 0.002), income (*p* = 0.002), and dietary habits (*p* < 0.001) **(Table ****S1****).**

## Distribution of responses to knowledge, attitudes, and practices

In the knowledge dimension, 78.64% of patients believed that brucellosis is curable and 65.84% of patients could correctly identify the transmission routes of brucellosis (**Table ****S2**).

In the attitude dimension, 23.37% of participants strongly agreed, and 46.98% agreed that they are concerned about the cost of treatment for brucellosis (A8). Conversely, 16.58% disagreed, and 1.51% strongly disagreed that brucellosis is a serious infectious disease (A1). Additionally, 4.02% disagreed, and 0.50% strongly disagreed that failing to actively treat brucellosis may lead to complications (A9) (**Table S3**).

Regarding the practices dimension, 6.28% reported rarely, and 7.04% reported never taking protective measures when handling animals (P3). Furthermore, 5.53% rarely, and 6.78% never share knowledge about brucellosis prevention with family and friends (P9). Lastly, 8.04% rarely, and 4.27% never avoid contact with animals that may be infected with *Brucella* (P6) (**Table S4**).

### Correlations between KAP

Correlation analysis showed that there were significant positive correlations between knowledge and attitudes (*r* = 0.332, *P* < 0.001) as well as practices (*r* = 0.378, *P* < 0.001). Also, there was a correlation between attitudes and practices (*r* = 0.350, *P* < 0.001) (Table 2).

**Table 2:** Correlation analysis.

	Knowledge	Attitudes	Practices
Knowledge	1		
Attitude	0.332 (*P* < 0.001)	1	
Practice	0.378 (*P* < 0.001)	0.350 (*P* < 0.001)	1

### Univariate and multivariate analysis of practices dimension

Multivariate linear regression showed that knowledge (β = 0.492, 95% CI: 0.319–0.664, *P* < 0.001), attitudes (β = 0.346, 95% CI: 0.214–0.478, *P* = 0.017), urban residence (β = 2.318, 95% CI: 1.075–3.561, *P* < 0.001), pastoral area residence (β = −1.249, 95% CI: −2.334 to −0.165, *P* = 0.024), and consumption of raw beef or mutton daily (β = −1.302, 95% CI: −2.374 to −0.230, *P* = 0.017) were independently associated with practices **(**Table 3**)**.

**Table 3:** Univariate and multivariate analysis for practices.

	Univariate		Multivariate	
	β (95%CI)	*P*	β (95%CI)	*P*
**Knowledge**	0.672(0.508–0.835)	< 0.001	0.492(0.319–0.664)	< 0.001
**Attitude**	0.472(0.342–0.603)	< 0.001	0.346(0.214–0.478)	0.017
**Gender**				
Male	−0.726(−1.840-0.388)	0.201		
Female	ref			
**Residence**				
Urban	2.885(1.764–4.007)	< 0.001	2.318(1.075–3.561)	< 0.001
Rural	ref		ref	
**Pastoral area residence**				
Yes	−1.357(−2.431- −0.283)	0.013	−1.249(−2.334- −0.165)	0.024
No	ref		ref	
**Occupations involving contact with animals**				
Yes	−1.427(−2.550- −0.304)	0.013	0.185(−0.977-1.347)	0.755
No	ref		ref	
**Education**				
Primary school or below	ref		ref	
Junior high school	0.539(−0.864-1.943)	0.450	0.314(−0.924-1.552)	0.618
Senior high school/technical secondary school	0.941(−0.744-2.625)	0.273	0.915(−0.576-2.406)	0.228
Associate degree	2.560(0.814–4.307)	0.004	1.372(−0.280-3.024)	0.103
Bachelor’s degree or above	3.076(1.392–4.760)	< 0.001	0.431(−1.419-2.281)	0.647
**Monthly income per capita**				
< 5000	ref		ref	
5000–10,000	2.245(0.988–3.503)	< 0.001	0.506(−0.715-1.726)	0.416
> 10,001	0.024(−2.490-2.538)	0.985	−2.328(−4.743-0.086)	0.059
**Raw beef or mutton in daily diet**				
Yes	−2.028(−3.211- −0.845)	< 0.001	−1.302(−2.374- −0.230)	0.017
No	ref		ref	

### SEM analysis

**Fig. 2:**
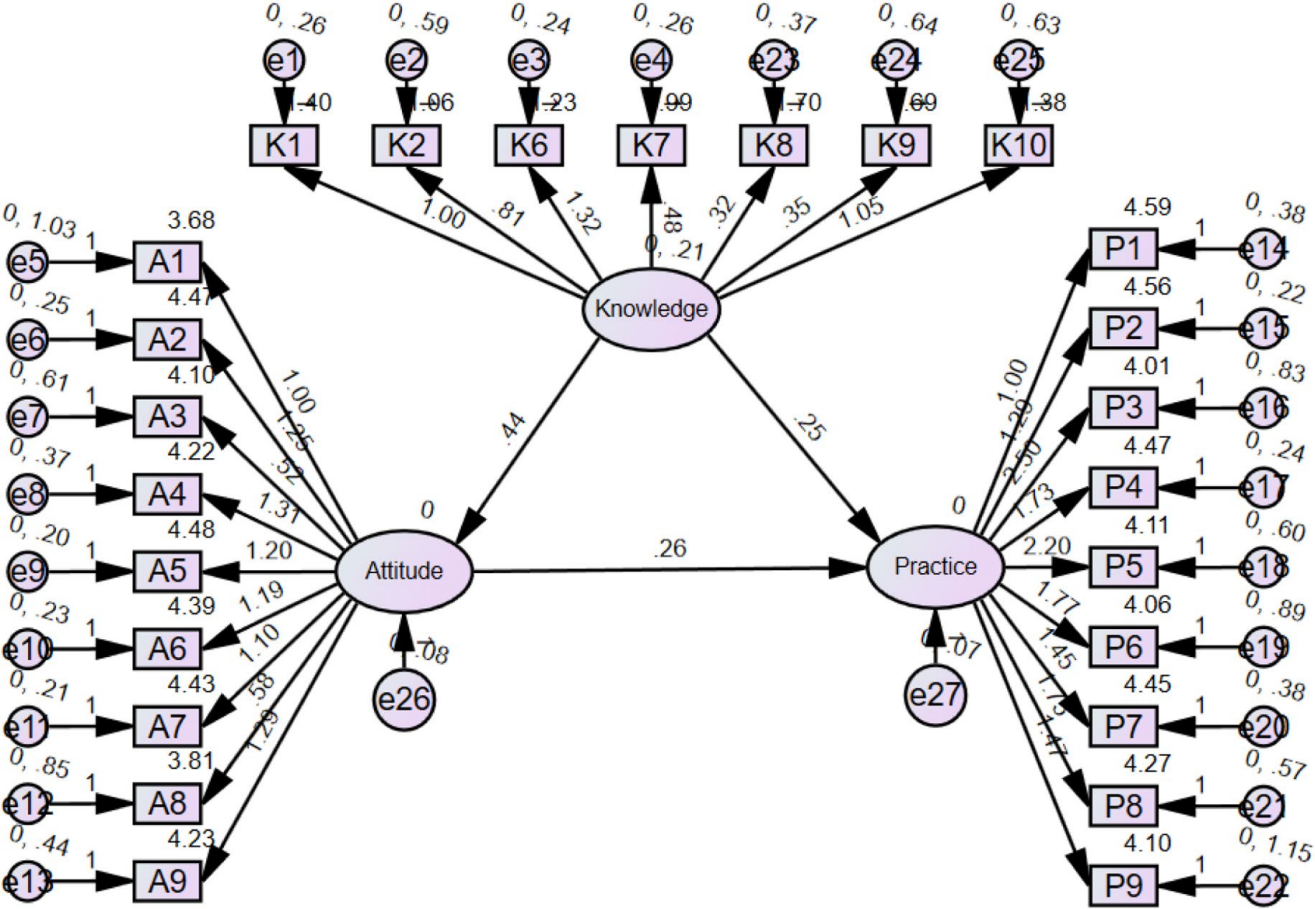
SEM model.

The SEM demonstrate a highly favorable model fit indices (CMIN/DF value: 2.416, IFI value: 0.846, TLI value: 0.829, and CFI value: 0.845), suggesting a well-fitting model (Table 4), and the results showed that the direct effect of knowledge on both attitudes (β = 0.579, *P* < 0.001) and practices (β = 0.351, *P* < 0.001), as well as of attitudes on practices (β = 0.270, *P* = 0.002), furthermore, knowledge indirectly affected practices through attitudes (β = 0.156, *P* = 0.010) (Table 5**and** Fig. 2).

**Table 4:** SEM fit indicators.

Model 1	Ref.	Measured results
CMIN/DF	1–3 excellent, 3–5 good	2.416
IFI	> 0.8 good	0.846
TLI	> 0.8 good	0.829
CFI	> 0.8 good	0.845

**Table 5:** SEM results.

Model paths	Standardized direct effects β (95%CI)	*P*	Standardized indirect effects β (95%CI)	*P*
Knowledge → Attitudes	0.579 (0.441–0.688)	< 0.001		
Knowledge → Practices	0.351 (0.217–0.491)	< 0.001	0.156 (0.053–0.242)	0.010
Attitudes → Practices	0.270 (0.084–0.409)	0.002		

## Discussion

Although patients with brucellosis showed relatively low knowledge scores, they exhibited positive attitudes and engaged in proactive health practices. Therefore, targeted educational interventions should be implemented to improve patients’ understanding. Enhancing awareness can strengthen positive attitudes and encourage preventive practices, ultimately supporting disease control and management. The SEM results showed that knowledge was associated with both attitudes and practices, and that attitudes were also associated with practices. An indirect association between knowledge and practices through attitudes was observed, which is consistent with the hypothesized relationships within the KAP framework^11,18,19^.

SEM revealed that knowledge significantly was associated with both attitudes and practices, and an indirect association through attitudes was observed^18,20^. This suggests that while knowledge is a key correlate of health behaviors, additional factors such as social norms, prior experiences, and structural barriers also play a role^18^. Understanding these relationships provides insight into potential intervention strategies to improve public health responses to brucellosis.

The knowledge deficits observed in this study align with findings from similar research in rural and agricultural communities, where awareness of zoonotic diseases remains low despite occupational exposure risks^19^. Many participants lacked understanding of brucellosis transmission, treatment, and preventive measures. For example, a substantial portion of the respondents did not recognize that brucellosis is a zoonotic disease, nor were they aware of its curability. This aligns with studies showing that low health literacy often results in delayed diagnosis and poor adherence to treatment regimens^21^. The correlation analysis confirmed a positive association between knowledge and both attitudes and practices, indicating that higher awareness was associated with improved behavioral responses. However, knowledge gaps were not uniformly distributed across demographic groups. Education level played a significant role in determining knowledge scores, but multivariate analysis was not associated between education and practices, suggesting that formal education alone may not be sufficient to drive behavioral change. In this study, knowledge, attitudes, urban residence, pastoral residence, and daily consumption of raw beef or mutton remained significant predictors of practices in the multivariate model.

Addressing these gaps requires targeted educational interventions that go beyond traditional informational campaigns. Community-based training, interactive workshops, and mobile health platforms have been shown to improve knowledge retention and behavioral adherence in similar settings^22^. Healthcare professionals should play an active role in disseminating accurate and accessible information, ensuring that high-risk groups, including livestock workers and rural residents, receive practical guidance tailored to their needs. In addition, integrating brucellosis education into routine veterinary services and agricultural extension programs could enhance disease prevention efforts among those at greatest risk^23^.

These findings provide context for understanding how patients’ limited knowledge contrasts with their generally positive attitudes toward brucellosis prevention.

While knowledge gaps were prominent, attitudes toward brucellosis prevention and treatment were generally positive. This discrepancy between limited knowledge and positive attitudes could potentially be explained by the direct experience of illness among these participants, which might have heightened their risk perception and treatment adherence despite lacking comprehensive disease information^22,23^. The high prevalence of occupational exposure (65.08%) in our sample suggests that many participants might have received practical safety instructions in their workplace without necessarily understanding the underlying disease mechanisms. Additionally, cultural factors in Xinjiang communities could plausibly contribute to compliance with medical advice as a social norm, which may help explain positive health behaviors even when theoretical knowledge is limited^24^. Most participants recognized the seriousness of the disease and the importance of adhering to medical advice. This trend aligns with findings from other infectious disease studies, where strong health beliefs contribute to better disease management outcomes^24,25^. The SEM results further support this, demonstrating that attitudes significantly were associated with practices, reinforcing the idea that beliefs and perceptions are related to preventive behaviors. However, the relatively high attitudes scores did not always translate into consistent preventive practices. Structural factors such as residence and daily dietary habits may also influence how individuals implement preventive practices. In the multivariate model, these factors were also independently associated with practice scores. This discrepancy suggests that while individuals may be aware of recommended behaviors, other factors such as resource availability, cultural norms, or risk perception may influence their actual implementation^26,27^. Therefore, addressing these inconsistencies between attitudes and practices requires reinforcing motivation and providing supportive environments for behavior change.

Encouraging positive health behaviors requires reinforcement strategies that build on existing attitudes. Peer education, patient support groups, and tailored communication strategies that emphasize real-life benefits can further strengthen positive beliefs and encourage consistent adherence to protective measures. Additionally, addressing misinformation and common misconceptions through trusted community figures, including healthcare providers and local leaders, could enhance message credibility and influence behavior on a broader scale^28,29^.

Despite favorable attitudes, gaps in practices remain, particularly concerning protective measures in occupational settings. While adherence to prescribed medication was high, preventive behaviors such as using protective gear when handling animals were inconsistent. This pattern has been observed in similar populations, where practical constraints such as financial limitations, lack of access to personal protective equipment, and workplace culture hinder the adoption of recommended safety measures^30,31^. The multivariate analysis showed that higher knowledge and more positive attitudes were associated with more proactive practices.

Improving adherence to preventive practices requires systemic changes. Expanding access to protective equipment and ensuring its affordability could remove key barriers to compliance. Occupational safety policies should be strengthened, particularly in high-risk professions, through workplace health and safety training. Additionally, embedding brucellosis prevention strategies within broader public health programs can ensure that interventions are sustainable and integrated into routine healthcare delivery. Collaborative efforts involving government agencies, healthcare institutions, and community organizations can create a more coordinated response, bridging gaps between policy and practices^32^.

The interconnections between knowledge, attitudes, and practices highlight the need for a comprehensive, multi-sectoral approach to brucellosis prevention and control. While knowledge serves as a foundational correlate of behavior, broader systemic factors such as healthcare accessibility, socio-economic conditions, and cultural perceptions may influence how individuals engage in preventive practices. Studies from other endemic regions indicate that successful interventions require an integration of education, policy changes, and community engagement^7,20^. Aligning public health strategies with the realities of high-risk populations ensures that interventions are both practical and effective.

Improving healthcare delivery and professional education is essential for long-term disease control. Embedding KAP assessments into routine public health surveillance can help identify gaps and inform targeted interventions. Strengthening interdisciplinary collaboration between medical professionals, veterinarians, and public health experts can enhance coordination efforts, ensuring a more holistic approach to brucellosis prevention. Additionally, leveraging digital tools and telemedicine could improve patient education and facilitate remote consultations, reducing delays in diagnosis and treatment^33^. Although the SEM framework is consistent with a causal model, these findings represent associations rather than confirmed causal relationships because of the cross-sectional design.

This study has several limitations. First, as a cross-sectional design, it cannot establish causal relationships between knowledge, attitudes, and practices among brucellosis patients. Second, the self-reported nature of the questionnaire may introduce recall and social desirability biases, as participants might have overreported favorable practices or attitudes, potentially affecting the accuracy of responses. Third, the use of convenience sampling may have introduced selection bias, as patients who were more health-literate, engaged in regular follow-up, or more motivated to participate could have been overrepresented. This potential bias may limit the representativeness of the sample and should be considered when interpreting the generalizability of the results. Fourth, the study was conducted in a single region (Xinjiang), which may limit the generalizability of the findings to broader populations with different socio-cultural and epidemiological contexts. Finally, as this study focused solely on laboratory-confirmed brucellosis patients without including a comparison group, the findings should be interpreted with caution when considering their uniqueness and broader applicability. Taken together, these limitations should be acknowledged when interpreting the findings and their implications for broader public health strategies.

In conclusion, patients with brucellosis showed low knowledge scores but displayed positive attitudes and proactive behaviors. Knowledge and attitudes significantly influence health-related behaviors. Therefore, targeted educational interventions are crucial for improving patients’ understanding. By increasing awareness and promoting positive attitudes, we can enhance the effectiveness of brucellosis prevention and control efforts.

## Supplementary Information

Below is the link to the electronic supplementary material.

Supplementary Material 1

Supplementary Material 2

## Data Availability

All data generated or analyzed during this study are included in this article and supplementary information files.
