# Supplementary material for: Knowledge, attitude, and practice regarding brucellosis among confirmed cases
Source: Sci Rep. 2025 Nov 28;15:45631. doi: 10.1038/s41598-025-30473-9 (PMC12753839; doi:10.1038/s41598-025-30473-9)
Supplement: Supplementary file 1 — Supplementary Material 1 [file 41598_2025_30473_MOESM1_ESM.docx]

| **Knowledge, Attitudes, and Practices toward Brucellosis among Brucellosis Patients** | |
| --- | --- |
| **Part 1 Basic Information** | |

| **Age, years** |
| --- |
| **Gender** |
| Male |
| Female |
| **Residence** |
| Urban |
| Rural |
| **Pastoral area residence** |
| Yes |
| No |
| **Occupations involving contact with animals** |
| Yes |
| No |
| **Education** |
| Primary school or below |
| Junior high school |
| Senior high school/technical secondary school |
| Associate degree |
| Bachelor’s degree or above |
| **Monthly income per capita** |
| <5000 |
| 5000-10000 |
| >10001 |
| **Raw beef or mutton in daily diet** |
| Yes |
| No |

| **Part 2 Knowledge** | | | |  |
| --- | --- | --- | --- | --- |
| **1. Brucellosis is a zoonotic infectious disease caused by *Brucella* bacteria. Humans can contract the disease through contact with excretions from infected animals or by consuming food products made from infected or diseased animals.** | **Unclear** | **Heard of it** | **Very familiar** | |
| **2. What is the pathogen of brucellosis?** | **Unclear** | **Heard of it** | **Very familiar** | |
| **3. Which of the following animals can be infected with Brucella?** | Correct | Wrong | Unclear | |
| **4. What are the transmission routes of brucellosis?** | Correct | Wrong | Unclear | |
| **5. What symptoms do humans exhibit when infected with brucellosis?** | Correct | Wrong | Unclear | |
| **6. Slaughterhouse workers, meat processing workers, and veterinarians are high-risk groups for brucellosis.** | **Unclear** | **Heard of it** | **Very familiar** | |
| **7. Can brucellosis be transmitted from person to person?** | **Unclear** | **Heard of it** | **Very familiar** | |
| **8. Is brucellosis curable?** | Yes | No | Unclear | |
| **9. Is there a vaccine for brucellosis in humans?** | Yes | No | Unclear | |
| **10. Have you ever been exposed to educational materials on brucellosis?** | Yes | No | Unclear | |

| **Part 3 Attitudes** | | | | | |
| --- | --- | --- | --- | --- | --- |
| **You believe that brucellosis is a serious infectious disease.** | a. Strongly agree | b. Agree | c. Neutral | d. Disagree | e. Strongly disagree |
| **You believe that timely medical treatment and following doctors’ advice are key to treating brucellosis.** | a. Strongly agree | b. Agree | c. Neutral | d. Disagree | e. Strongly disagree |
| **You believe that brucellosis is curable.** | a. Strongly agree | b. Agree | c. Neutral | d. Disagree | e. Strongly disagree |
| **You believe that consuming undercooked beef, lamb, dried meat, or air-dried meat may lead to brucellosis infection.** | a. Strongly agree | b. Agree | c. Neutral | d. Disagree | e. Strongly disagree |
| **You believe that society should provide more information and awareness campaigns on brucellosis because the public should be more informed about the disease and its prevention.** | a. Strongly agree | b. Agree | c. Neutral | d. Disagree | e. Strongly disagree |
| **You believe that dairy products must be heated before consumption.** | a. Strongly agree | b. Agree | c. Neutral | d. Disagree | e. Strongly disagree |
| **You believe that meat should be purchased only if it has passed quarantine inspection.** | a. Strongly agree | b. Agree | c. Neutral | d. Disagree | e. Strongly disagree |
| **You are concerned about the cost of treatment for brucellosis.** | a. Strongly agree | b. Agree | c. Neutral | d. Disagree | e. Strongly disagree |
| **You believe that failure to actively treat brucellosis may result in complications.** | a. Strongly agree | b. Agree | c. Neutral | d. Disagree | e. Strongly disagree |

| **Part 4 Practices** | | | | | |
| --- | --- | --- | --- | --- | --- |
| **I take my medication as prescribed by my doctor.** | a. Always | b. Often | c. Sometimes | d. Rarely | e. Never |
| **I maintain good dietary hygiene by avoiding raw meat and only consuming thoroughly cooked meat.** | a. Always | b. Often | c. Sometimes | d. Rarely | e. Never |
| **When handling animals, I take protective measures (e.g., wearing rubber gloves, masks, work clothes).** | a. Always | b. Often | c. Sometimes | d. Rarely | e. Never |
| **After handling animal products or being in environments where *Brucella* may be present, I wash my hands promptly.** | a. Always | b. Often | c. Sometimes | d. Rarely | e. Never |
| **I regularly clean and disinfect items or environments that may be contaminated.** | a. Always | b. Often | c. Sometimes | d. Rarely | e. Never |
| **I avoid contact with animals that may be infected with *Brucella*, such as sheep, cattle, or pigs.** | a. Always | b. Often | c. Sometimes | d. Rarely | e. Never |
| **I ensure that dairy products are properly pasteurized and do not consume raw, unpasteurized milk.** | a. Always | b. Often | c. Sometimes | d. Rarely | e. Never |
| **When purchasing food, I check for safety inspection and certification.** | a. Always | b. Often | c. Sometimes | d. Rarely | e. Never |
| **I share knowledge about brucellosis prevention with my family and friends.** | a. Always | b. Often | c. Sometimes | d. Rarely | e. Never |
