# Supplementary material for: Knowledge, attitude, and practice regarding brucellosis among confirmed cases
Source: Sci Rep. 2025 Nov 28;15:45631. doi: 10.1038/s41598-025-30473-9 (PMC12753839; doi:10.1038/s41598-025-30473-9)
Supplement: Supplementary file 2 — Supplementary Material 2 [file 41598_2025_30473_MOESM2_ESM.docx]

**Supplementary table 1 Knowledge, Attitudes, and Practices scores across various baseline characteristics**

|  | **Knowledge, mean ± SD** | **P** | **Attitudes, mean ± SD** | **P** | **Practices，mean ± SD** | **P** |
| --- | --- | --- | --- | --- | --- | --- |
|  |  |  |  |  |  |  |
| **Total** | 8.45±3.06 |  | 37.81±3.89 |  | 38.62±5.47 |  |
| **Age, years** |  |  |  |  |  |  |
| **Gender** |  | 0.241 |  | 0.641 |  | 0.285 |
| Male | 8.36±2.87 |  | 37.70±3.97 |  | 38.35±5.67 |  |
| Female | 8.61±3.35 |  | 38.00±3.77 |  | 39.07±5.10 |  |
| **Residence** |  | 0.147 |  | 0.465 |  | **<0.001** |
| Urban | 8.69±3.20 |  | 37.48±4.39 |  | 40.58±5.00 |  |
| Rural | 8.34±2.99 |  | 37.96±3.63 |  | 37.70±5.44 |  |
| **Pastoral area residence** |  | **0.033** |  | **0.005** |  | **0.003** |
| Yes | 8.80±3.00 |  | 38.47±3.35 |  | 37.89±5.10 |  |
| No | 8.15±3.08 |  | 37.25±4.23 |  | 39.24±5.70 |  |
| **Occupations involving contact with animals** |  | 0.191 |  | 0.68 |  | **0.009** |
| Yes | 8.37±2.86 |  | 37.88±3.73 |  | 38.12±5.44 |  |
| No | 8.60±3.40 |  | 37.68±4.18 |  | 39.55±5.41 |  |
| **Education** |  | **<0.001** |  | 0.13 |  | **0.002** |
| Primary school or below | 7.84±3.05 |  | 38.05±3.74 |  | 37.53±5.95 |  |
| Junior high school | 8.17±2.86 |  | 37.43±3.94 |  | 38.07±5.33 |  |
| Senior high school/technical secondary school | 8.37±2.91 |  | 37.27±3.80 |  | 38.47±5.08 |  |
| Associate degree | 8.30±3.24 |  | 37.58±4.03 |  | 40.09±4.64 |  |
| Bachelor’s degree or above | 10.39±2.72 |  | 38.76±3.99 |  | 40.61±4.71 |  |
| **Monthly income per capita** |  | **<0.001** |  | 0.239 |  | **0.002** |
| <5000 | 7.97±2.98 |  | 37.64±3.83 |  | 38.08±5.51 |  |
| 5000-10000 | 9.59±2.76 |  | 38.34±3.99 |  | 40.33±4.72 |  |
| >10001 | 9.89±3.75 |  | 37.63±4.31 |  | 38.11±6.77 |  |
| **Raw beef or mutton in daily diet** |  | 0.522 |  | **0.005** |  | **<0.001** |
| Yes | 8.11±3.80 |  | 36.93±4.06 |  | 37.16±4.96 |  |
| No | 8.58±2.71 |  | 38.15±3.77 |  | 39.19±5.56 |  |

**Supplementary table 2. Knowledge dimension**

| **Items, n (%)** | **Unclear** | **Heard of it** | **Very familiar** |
| --- | --- | --- | --- |
| **1. Brucellosis is a zoonotic infectious disease caused by *Brucella* bacteria. Humans can contract the disease through contact with excretions from infected animals or by consuming food products made from infected or diseased animals.** | 204 (51.26%) | 148 (37.19%) | 46 (11.56%) |
| **2. What is the pathogen of brucellosis?** | 158 (39.70%) | 107 (26.88%) | 133 (33.42%) |
| **6. Slaughterhouse workers, meat processing workers, and veterinarians are high-risk groups for brucellosis.** | (6.03%) | 506 (92.50%) | 8 (1.46%) |
|  |  |  |  |
| **7. Can brucellosis be transmitted from person to person?** | 59 (14.82%) | 277 (69.60%) | 62 (15.58%) |
|  | Correct | Wrong | Unclear |
| **3. Which of the following animals can be infected with Brucella?** | 66(16.58%) | 310(77.90%) | 22(5.53%) |
| **4. What are the transmission routes of brucellosis?** | 261(65.58%) | 87(21.86%) | 50(12.56%) |
| **5. What symptoms do humans exhibit when infected with brucellosis?** | 206(51.76%) | 163(40.95%) | 29(7.29%) |
|  | Yes | No | Unclear |
| **8. Is brucellosis curable?** | 313 (78.64%) | 49 (12.31%) | 36 (9.05%) |
| **9. Is there a vaccine for brucellosis in humans?** | 90 (22.61%) | 95 (23.87%) | 213 (53.52%) |
| **10. Have you ever been exposed to educational materials on brucellosis?** | 275(69.10%) | 78 (19.60%) | 45 (11.31%) |

**Supplementary table 3. Attitude dimension**

| **Items, n (%)** | **Strongly agree** | **Agree** | **Neutral** | **Disagree** | **Strongly disagree** |
| --- | --- | --- | --- | --- | --- |
| 1. **You believe that brucellosis is a serious infectious disease.** | 101 (25.38%) | 147 (36.93%) | 78 (19.60%) | 66 (16.58%) | 6(1.51%) |
| 1. **You believe that timely medical treatment and following doctors’ advice are key to treating brucellosis.** | 211 (53.02%) | 172 (43.22%) | 8 (2.01%) | 4 (1.01%) | 3 (0.75%) |
| 1. **You believe that brucellosis is curable.** | 126 (31.66%) | 211 (53.02%) | 38 (9.55%) | 22 (5.53%) | 1(0.25%) |
| 1. **You believe that consuming undercooked beef, lamb, dried meat, or air-dried meat may lead to brucellosis infection.** | 147 (36.93%) | 207 (52.01%) | 32 (8.04%) | 8(2.01%) | 4(1.01%) |
| 1. **You believe that society should provide more information and awareness campaigns on brucellosis because the public should be more informed about the disease and its prevention.** | 211 (53.02%) | 172 (43.22%) | 13 (3.27%) | 0 | 2(0.50%) |
| 1. **You believe that dairy products must be heated before consumption.** | 180 (45.23%) | 198 (49.75%) | 16 (4.02%) | 3 (0.75%) | 1(0.25%) |
| 1. **You believe that meat should be purchased only if it has passed quarantine inspection.** | 190(47.74%) | 191(47.99%) | 15(3.77%) | 2(0.50%) | 0 |
| 1. **You are concerned about the cost of treatment for brucellosis.** | 93(23.37%) | 187(46.98%) | 68(17.09%) | 48(12.06%) | 2(0.50%) |
| 1. **You believe that failure to actively treat brucellosis may result in complications.** | 161(40.45%) | 188(47.24%) | 31(7.79%) | 16(4.02%) | 2(0.50%) |

**Supplementary table 4. Practice dimension**

| **Items, n (%)** | **Always** | **Often** | **Sometimes** | **Rarely** | **Never** |
| --- | --- | --- | --- | --- | --- |
| 1. **I take my medication as prescribed by my doctor.** | 266(66.83%) | 111 (27.89%) | 13 (3.27%) | 4 (1.01%) | 4 (1.01%) |
| 1. **I maintain good dietary hygiene by avoiding raw meat and only consuming thoroughly cooked meat.** | 245 (61.56%) | 139 (34.92%) | 9 (2.26%) | 3(0.75%) | 2(0.50%) |
| 1. **When handling animals, I take protective measures (e.g., wearing rubber gloves, masks, work clothes).** | 192 (48.24%) | 99 (24.87%) | 54 (13.57%) | 25 (6.28%) | 28(7.04%) |
| 1. **After handling animal products or being in environments where *Brucella* may be present, I wash my hands promptly.** | 229(57.54%) | 141 (35.43%) | 16 (4.02%) | 9 (2.26%) | 3(0.75%) |
| 1. **I regularly clean and disinfect items or environments that may be contaminated.** | 178(44.72%) | 140(35.18%) | 40(10.05%) | 26(6.53%) | 14(3.52%) |
| 1. **I avoid contact with animals that may be infected with *Brucella*, such as sheep, cattle, or pigs.** | 173(43.47%) | 140(35.18%) | 36(9.05%) | 32(8.04%) | 17(4.27%) |
| 1. **I ensure that dairy products are properly pasteurized and do not consume raw, unpasteurized milk.** | 225(56.53%) | 150(37.69%) | 8(2.01%) | 9(2.26%) | 6(1.51%) |
| 1. **When purchasing food, I check for safety inspection and certification.** | 206(51.76%) | 131(32.91%) | 32(8.04%) | 22(5.53%) | 7(1.76%) |
| 1. **I share knowledge about brucellosis prevention with my family and friends.** | 194(48.74%) | 125(31.41%) | 30(7.54%) | 22(5.53%) | 27(6.78%) |
